# Sonographic findings in coronavirus disease-19 associated liver damage

**DOI:** 10.1371/journal.pone.0244781

**Published:** 2021-02-19

**Authors:** Jakob Spogis, Florian Hagen, Wolfgang M. Thaiss, Tatjana Hoffmann, Nisar Malek, Konstantin Nikolaou, Christoph P. Berg, Stephan Singer, Hans Bösmüller, Florian Kreth, Sascha Kaufmann

**Affiliations:** 1 Department of Diagnostic and Interventional Radiology, Eberhard-Karls-University, Tübingen, Germany; 2 Department of Nuclear Medicine, University Hospital Ulm, Ulm, Germany; 3 Internal Medicine, Eberhard-Karls-University, Tübingen, Germany; 4 Pathology, Eberhard-Karls-University, Tübingen, Germany; National Institute for Infectious Diseases Lazzaro Spallanzani-IRCCS, ITALY

## Abstract

**Purpose:**

This study was conducted to evaluate the role of liver sonography in patients with coronavirus disease 2019 (COVID-19) and elevated liver enzymes.

**Materials and methods:**

In this retrospective study, patients tested positive for SARS-CoV-2 in our emergency ward between January 01 and April 24, 2020 and elevated liver enzymes were included (Cohort 1). Additionally, the local radiology information system was screened for sonographies in COVID-19 patients at the intensive care unit in the same period (Cohort 2). Liver sonographies and histologic specimen were reviewed and suspicious findings recorded. Medical records were reviewed for clinical data. Ultrasound findings and clinical data were correlated with severity of liver enzyme elevation.

**Results:**

Cohort 1: 126 patients were evaluated, of which 47 (37.3%) had elevated liver enzymes. Severity of liver enzyme elevation was associated with death (p<0.001). 8 patients (6.3%) had suspicious ultrasound findings, including signs of acute hepatitis (n = 5, e.g. thickening of gall bladder wall, hepatomegaly, decreased echogenicity of liver parenchyma) and vascular complications (n = 4). Cohort 2: 39 patients were evaluated, of which 14 are also included in Cohort 1. 19 patients (48.7%) had suspicious ultrasound findings, of which 13 patients had signs of acute hepatitis and 6 had vascular complications. Pathology was performed in 6 patients. Predominant findings were severe cholestasis and macrophage activation.

**Conclusion:**

For most hospitalized COVID-19 patients, elevated liver enzymes cause little concern as they are only mild to moderate. However, in severely ill patients bedside sonography is a powerful tool to reveal different patterns of vascular, cholestatic or inflammatory complications in the liver, which are associated with high mortality. In addition, macrophage activation as histopathologic correlate for a hyperinflammatory syndrome seems to be a frequent complication in COVID-19.

## Introduction

In December 2019, the novel coronavirus (SARS-CoV-2) broke out in Wuhan, China. The associated disease, Coronavirus Disease 2019 (COVID-19), is typically characterized by the symptoms of a viral pneumonia, which may evolve to respiratory failure. With ongoing worldwide spread, more information about the incidence of elevated liver enzymes during the infection is gained. Increased AST and ALT was found in 21 and 22% of patients respectively [1], especially in severely ill patients. Nevertheless clinically significant acute liver damage seems to remain rare [2]. More often, the lungs, heart and kidneys are harmed in multiorgan failure [3].

The exact mechanism of liver damage in COVID-19 patients is unclear. In a biopsy specimen of a deceased patient with travel history to Wuhan, moderate microvascular steatosis and mild lobular and portal activation was seen, caused either by the infection or by drug toxicity [4]. Possible mechanisms of liver injury are: (a) Immune mediated damage due to severe inflammatory response, (b) direct cytotoxicity due to active viral replication, (c) anoxia in respiratory failure, (d) drug induced liver injury, or (e) reactivation of pre-existing liver disease [5].

Despite its high prevalence, no systematic investigation of the imaging workup of elevated transaminases was performed so far. This study aims to clarify the role of sonography because of its high usability in critically ill patients and intensive care units (ICU).

## Material and methods

### Institutional board approval

This retrospective single-center study was approved by the institutional ethics committee of the University of Tuebingen under the approval number 543/2020BO. Written informed consent was waived in order to obtain a representative study population in the retrospective analysis. The study was conducted in accordance with the current version of the Declaration of Helsinki.

### Patients

Two cohorts with partial overlap were examined in this retrospective study:

*Cohort 1*: Patients who were tested positive for SARS-CoV-2 in the emergency department of our hospital between January 01 and April 24, 2020 were screened for elevated liver enzymes. Patients were assigned to three groups according to the highest measured value of alanine aminotransferase (ALT):

1. normal ALT,
2. mild to moderate elevation defined as ALT < 10 times upper normal limit (UNL),
3. severe elevation, defined as ALT > 10 times UNL.

Patients of group 2 and 3 were followed with regard to documented sonographies, MRI and histologic specimen of the liver. The maximum values of ALT, aspartate aminotransferase (AST), gamma-glutamyl transferase (GGT), alkaline phosphatase (ALP) and total bilirubin were recorded.

*Cohort 2*: In a second analysis, the local radiology information system (RIS) was screened for sonographies performed at the ICU of our hospital in the same period as Cohort 1 as diagnostic workup for elevated liver enzymes in COVID-19 patients. All patients had positive test for SARS-CoV-2 in our institution. Patients were assigned to the above-mentioned groups and followed for histologic specimen and MRI for comparison with ultrasound findings. Maximum values of the same laboratory results as Cohort 1 were recorded.

### Sonography

Every patient admitted to our hospital with the diagnosis of COVID-19 and elevated liver enzymes received abdominal sonography during the inpatient treatment. Sonographies at ICU were performed using a Philips HD 11 XE or CX50 (Philips medical systems, Hamburg, Germany) with documentation in the local Picture Archiving and Communication System (PACS). Radiologists with a minimum of 3 years of clinical ultrasound experience performed the examinations at bedside. During the examination the physician wore a disposable cap, a FFP2-mask, protective suit and disposable latex gloves. None of the physicians got infected during the study. The liver and gall bladder were examined transcostally and subcostally with the patient in supine position.

Patients at the infection ward received sonography by the ward using a Philips Sparq (Philips medical systems, Hamburg, Germany).

### Image analysis

The ultrasound images were reevaluated by two physicians with 3 and 14 years of experience in clinical ultrasound. Images were analyzed with regard to signs of hepatomegaly, echogenicity of the liver compared with the kidneys and the intrahepatic bile ducts (also known as starry sky appearance in case of decreased echogenicity), cholestasis, flow signal of the portal vein, hepatic artery and veins, thickening of the gallbladder wall, cholecystolithiasis and ascites. In case of repeated examination the most suspicious findings were recorded.

### Statistical analysis

JMP 14.2.0 (SAS Institute Inc.; Cary, NC) was used for statistical analysis. Measurement data were presented as mean +/- standard deviation and analyzed using t-test, chi-square test and Fisher’s exact test. After verification of Gaussian distribution of every parameter by the Shapiro-Wilk test, we opted for a unpaired t-test. A p-value of less than 0.05 was considered statistically significant.

## Results

### Clinical data

**Fig 1:**
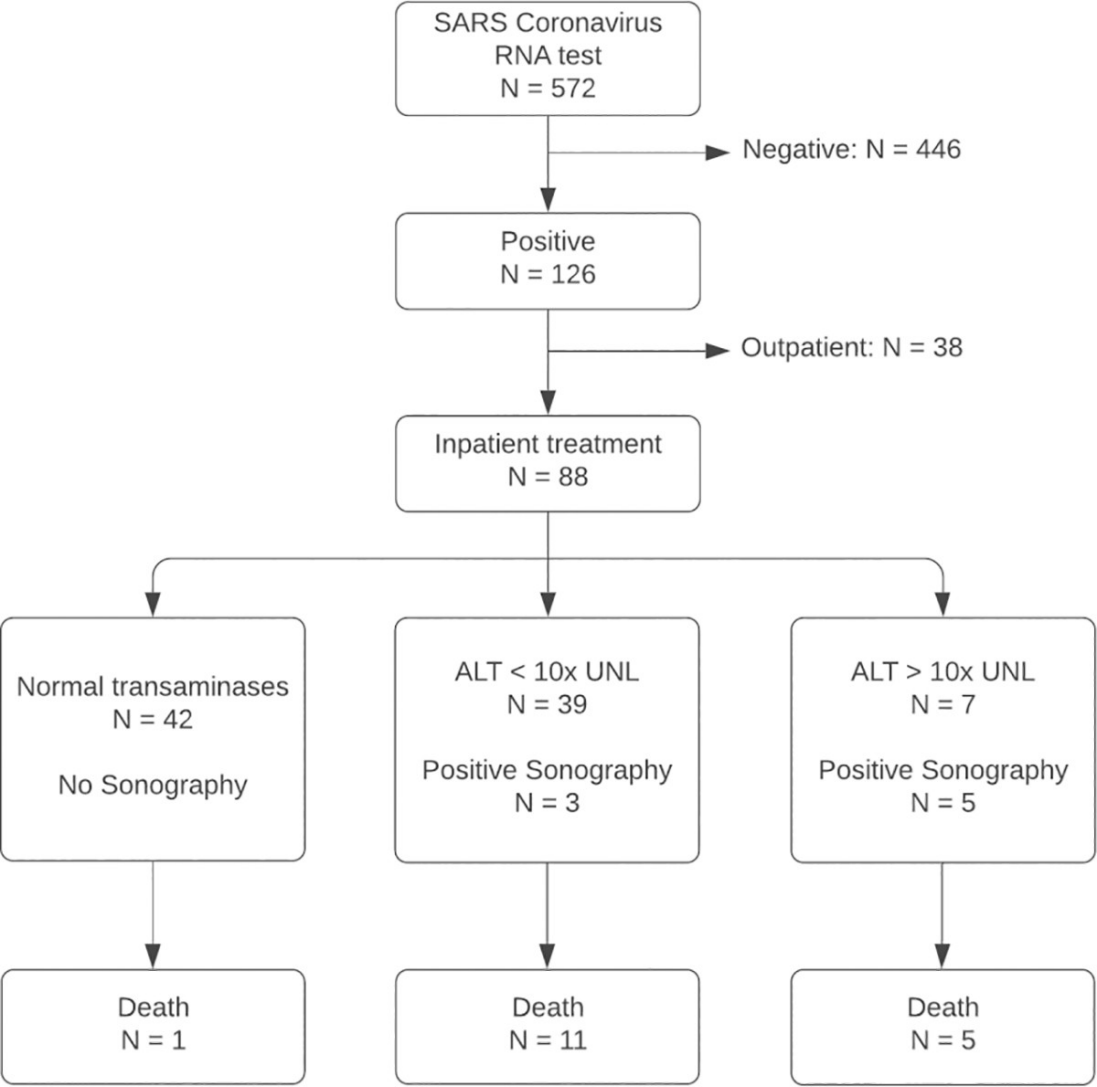
Procedure for research subject selection of cohort 1. All patients positively tested for SARS Coronavirus RNA were screened for elevated liver enzymes. ALT, alanine aminotransferase; UNL, upper normal limit.

**Fig 2:**
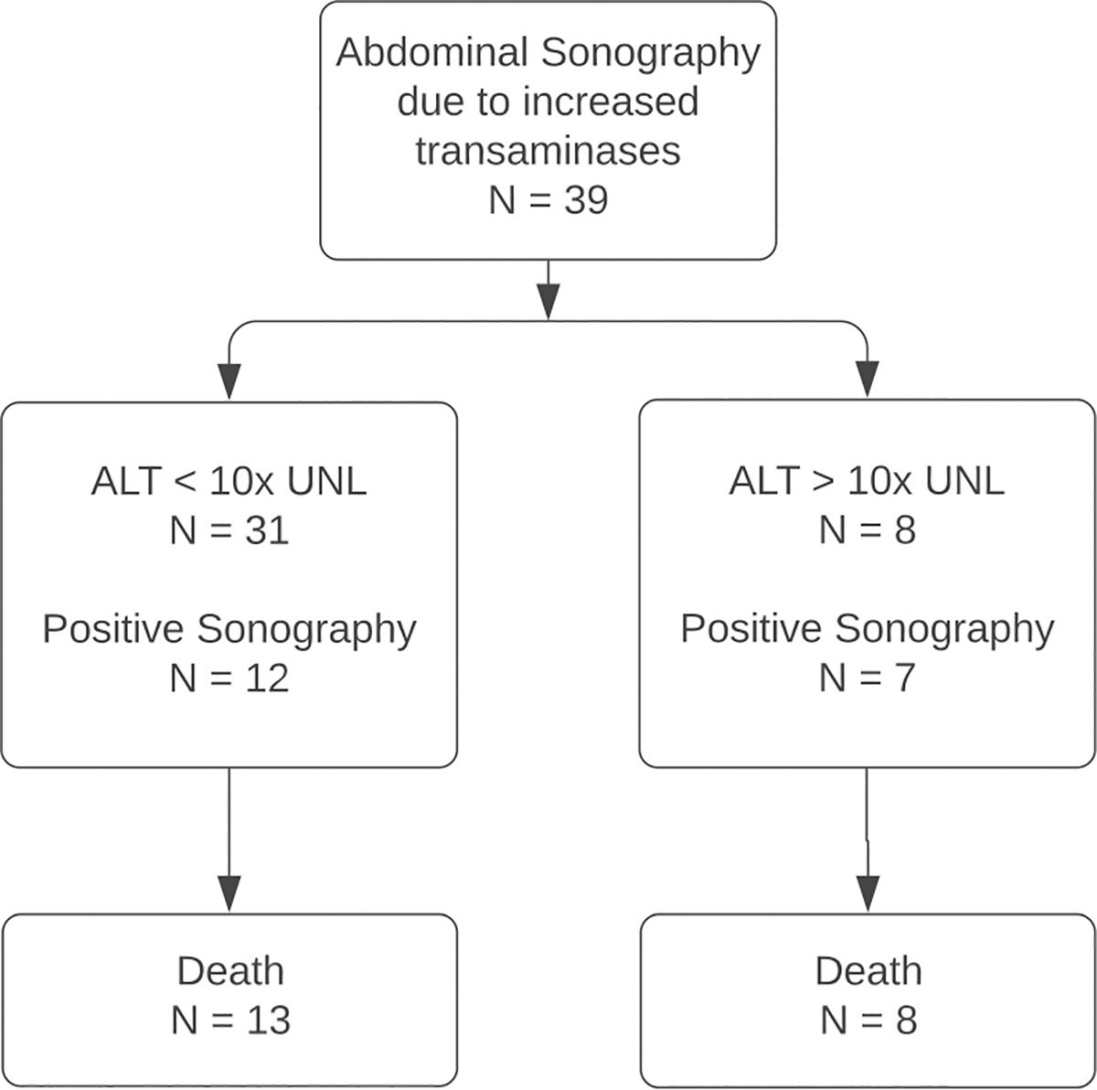
Procedure for research subject selection of cohort 2. The local radiology information system (RIS) was screened for abdominal sonographies at the intensive care unit for diagnostic workup of elevated liver enzymes. ALT, alanine aminotransferase; UNL, upper normal limit.

The flowchart of research subject selection and follow-up is shown in Fig 1 for cohort 1 and in Fig 2 for cohort 2. 14 patients are included in both cohorts. No patient had previously known liver disease such as viral hepatitis, liver cirrhosis or history of alcohol abuse.

*Cohort 1*: 126 patients were tested positive for SARS-CoV-19 in the emergency department (63 male and 63 female) with no significant difference in age between male and female patients (p = 0.15). 88 (69.8%, 88/126) received inpatient treatment, 36 (28.6%, 36/126) were admitted to ICU. 39 (31.0%, 39/126) were assigned to group 2 (mild to moderate elevation of liver enzymes, 7 (5.6%, 5/126) were assigned to group 3. In group 2, 23 (59.0%, 23/39) were male, in group 3, 6 (85.7%, 6/7) were male. 17 (13.5%, 17/126) patients died, of which 1 (1.3%, 1/80) in group 1, 11 (28.2%, 11/39) in group 2 and 5 (71.4%, 5/7) in group 3 (χ^2^ (2) = 37.64, p < 0.001).

*Cohort 2*: 39 patients at ICU received sonography of the liver, of which 14 are also included in cohort 1. 31 (79.5%, 31/39) patients were assigned to group 2 (mild to moderate elevation of transaminases), of which 13 (42%, 13/31) died. 8 (20.5%, 8/39) were assigned to group 3, all of which died (χ^2^ (1) = 7.77, p = 0.005).

Patient characteristics and laboratory results of both cohorts are shown in Table 1.

**Table 1:** Summary of patient characteristics and laboratory results.

	Cohort 1 (N = 126)			Cohort 2 (N = 39) ***	
	Group 1	Group 2	Group 3	Group 2	Group 3
	no elevation	ALT < 10x UNL	ALT > 10x UNL	ALT < 10x UNL	ALT > 10x UNL
**N (% of total cohort)**	80 (63.5)	39 (31.0)	7 (5.6)	31 (79.5)	8 (20.5)
**Male**	34 (42.5)	23 (59.0)	6 (85.7)	26 (83.9)	7 (87.5)
■ Age (Mean/SD)	60/18	77/9	68/14	64/15	70/16
**Female**	46 (57.5)	16 (41.0)	1 (14.3)	5 (16.1)	1 (12.5)
■ Age (Mean/SD)	60/20	68/20	72 *	70/16	80 *
**Inpatient treatment**	42 (52.5)	39 (100)	7 (100)	31 (100)	8 (100)
**ICU treatment**	1 (1.3)	28 (71.8)	7 (100)	31 (100)	8 (100)
**Death**	1 (1.3)	11 (28.2)	5 (71.4)	13 (41.9)	8 (100)
**Positive sonography**		3 (7.7)	5 (71.4)	12 (38.7)	7 (87.5)
**Max. lab parameter (Mean/SD):**					
AST	**	191/140	3302/2975	324/222	3754/2576
ALT	**	131/73	1254/741	166/101	1263/740
ALP	**	208/167	656/385	404/331	680/425
GGT	**	329/394	1187/928	824/764	1213/906
Total Bilirubin	**	3.0/4.9	7.5/7.9	8.2/11.0	12.4/13.1

ALT, alanine aminotransferase; AST, aspartate aminotransferase; GGT, gamma-glutamyl transferase; ALP, alkaline phosphatase; UNL, upper normal limit.

* One patient only.

** Parameters in normal range or not measured.

*** 14 patients are also part of cohort 1 (10 in group 2, 4 in group 3).

### Sonography findings

**Fig 3:**
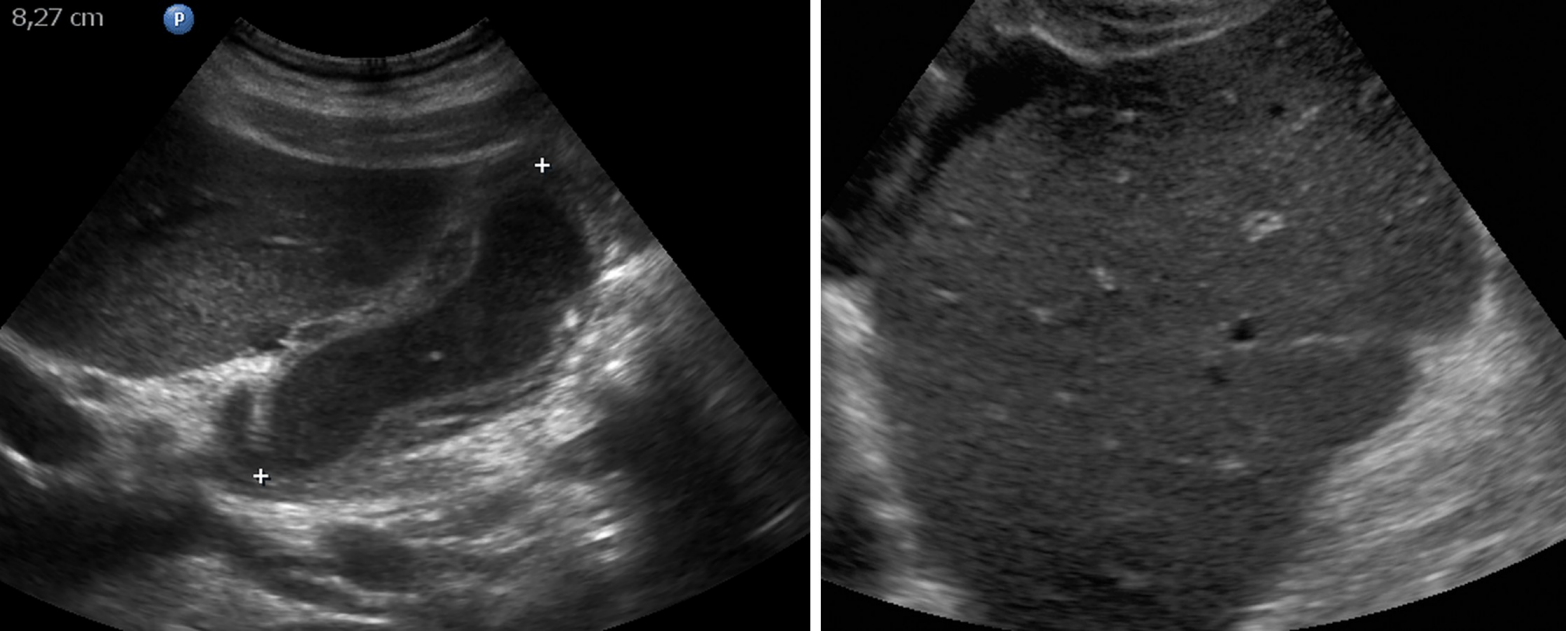
Signs of acute hepatitis. (A) Pronounced thickening of the gallbladder wall, filled with sludge. This represents a typical sonographic finding in our study. (B) Indicated starry sky appearance of the liver with decreased echogenicity of liver parenchyma and pronounced bile ducts.

**Fig 4:**
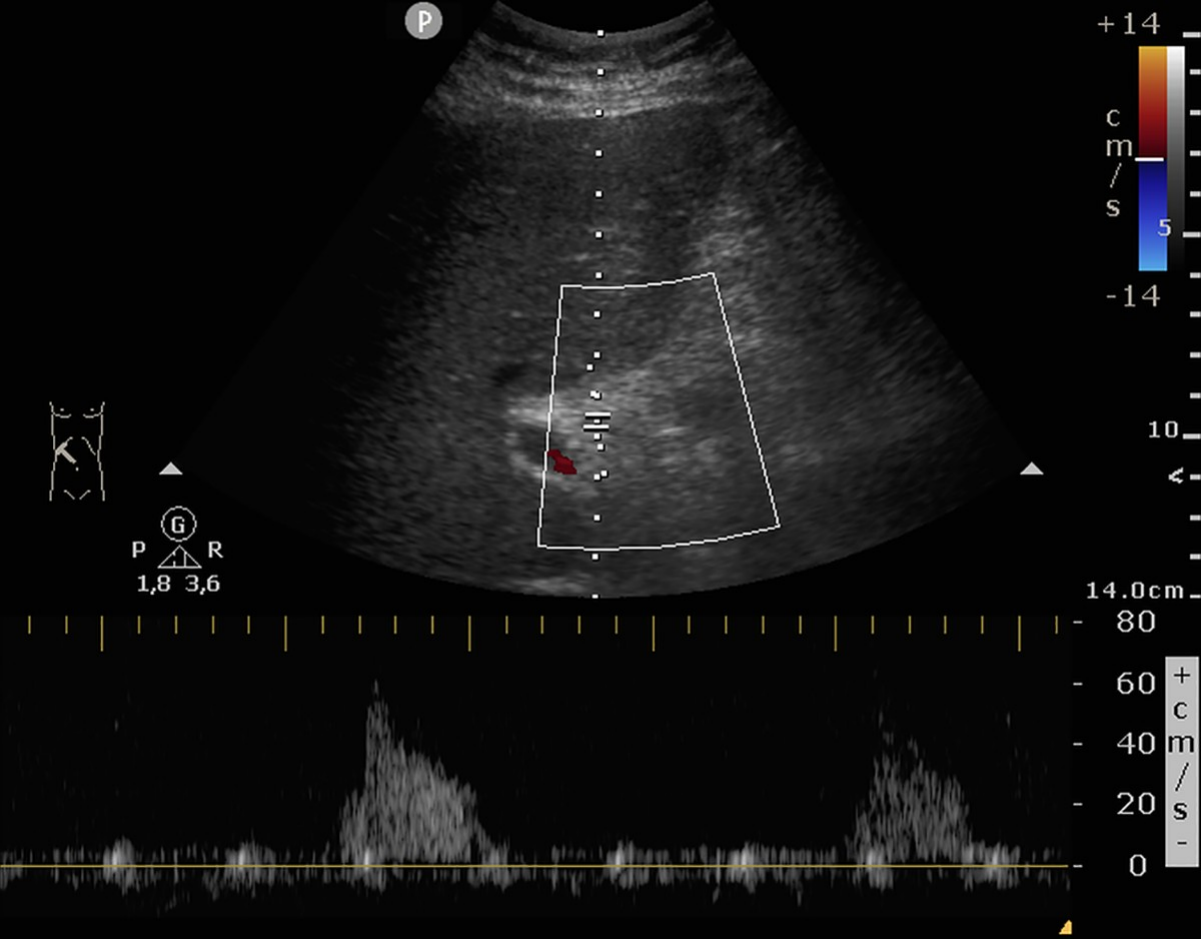
Vascular complications in the liver. In this patient with severe deterioration of liver function only residual arterial Doppler signal could be found in the porta hepatis. The patient died the same day.

**Fig 5:**
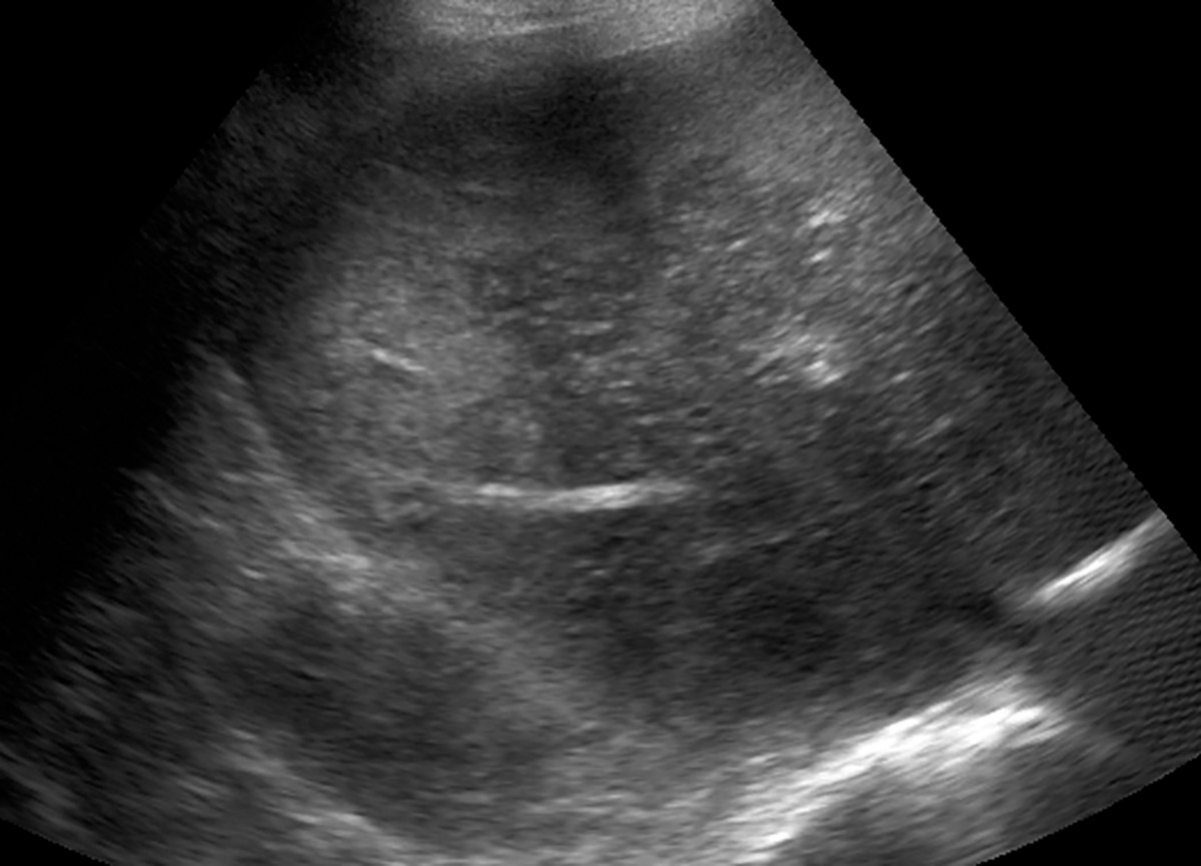
Vascular complications in the liver. In two cases, inhomogeneous echogenicity of the liver parenchyma was found. Autopsy confirmed the suspected diagnosis of liver necrosis in this case.

*Cohort 1*: In group 2, 3 (7.7%, 3/39) patients had positive sonography. All of them showed signs of acute hepatitis (thickening of gall bladder wall, hepatomegaly, decreased echogenicity of the liver, Fig 3). In one patient, additionally no Doppler signal was found in the hepatic artery (Fig 4). In group 3, 5 (71.4%, 5/7) had positive sonography. Two patients had signs of acute hepatitis, three showed signs of a vascular complication: two had no detectible Doppler signal of the hepatic artery, one showed inhomogeneous liver parenchyma as a sign of tissue necrosis (Fig 5). Positive sonography was not significantly associated with severity of liver enzyme elevation (p = 0.07) or death (p = 0.2).

*Cohort 2*: 12 (38.7%, 12/31) patients of group 2 had positive sonography (10x signs of acute hepatitis, one with inhomogeneous liver tissue, one with no detectable Doppler signal of the hepatic artery), of which two are also included in cohort 1. In group 3, 7 (87.5%, 7/8) had positive sonography (3x signs of acute hepatitis, 4x signs of vascular complication), of which four are also included in cohort 1. Positive sonography was associated with severity of liver enzyme elevation (p = 0.018) and death (p = 0.006).

Sonographic findings are summarized for both cohorts in Table 2.

**Table 2:** Summary of ultrasound findings matched for cohorts 1 and 2.

	Group 2	Group 3
	ALT < 10x UNL	ALT > 10x UNL
**Echogenicity of the liver**		
Decreased	2	0
Inhomogeneous	1	1
**Rounded liver angle / Hepatomegaly**	5	2
**Cholestasis**	0	0
**Not detectible Hepatic artery**	1	3
**Thickened gallbladder wall**	10	4
**Gallstones**	1	1
**Sludge**	7	2
**Ascites**	5	1

ALT, alanine aminotransferase; UNL, upper normal limit.

### Histology and other imaging findings

**Fig 6:**
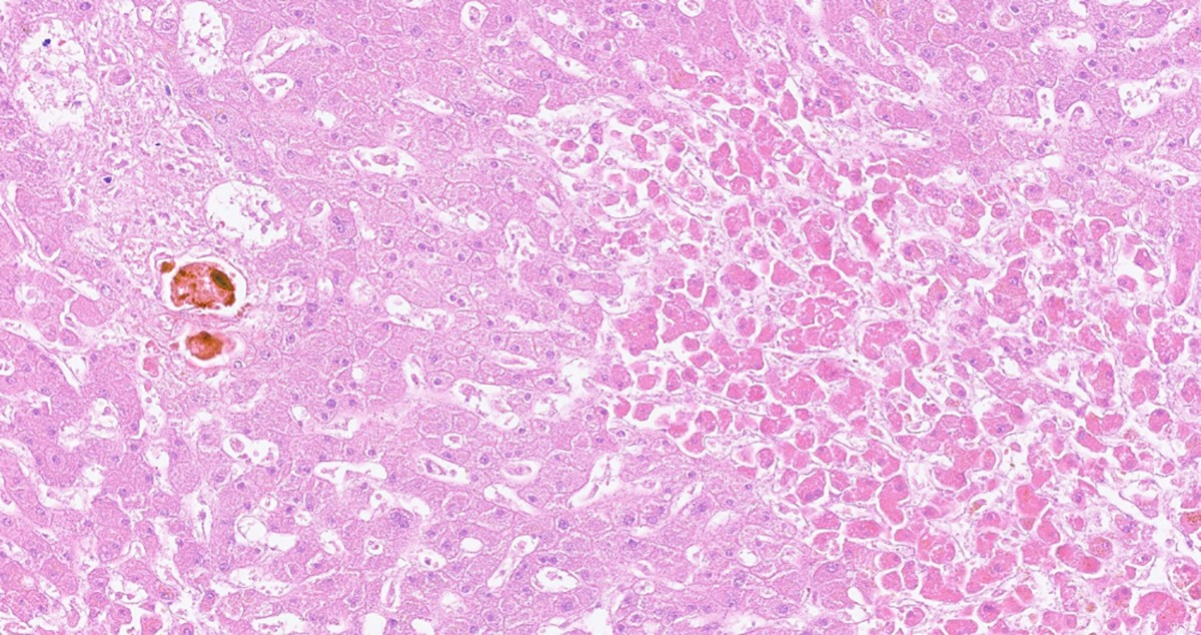
Histologic specimen of the liver. Progressive centroacinar hypoxic damage (right) and conspicuous cholestasis in adjacent portal field. HE x200.

Five patients with positive sonography received an autopsy. The predominant finding was severe cholestasis, found in four patients (Fig 6). Macrophage activation was found in three patients. Table 3 summarizes the histologic findings and their correlate in sonography.

**Table 3:** Summary of histologic findings with corresponding ultrasound findings.

	Sonography	Autopsy: Macroscopic	Histology
1	Inhomogeneous liver echogenicity	Hepatomegaly, Gallbladder unsuspicious	Centroacinary necrosis; massive cholestasis; macrophage activation; no inflammation
2	Hepatic artery not detectable; thickened gallbladder; ascites	Hepatomegaly; ascites	Normal structure; no necrosis; macrophage activation
3	Hepatomegaly; thickened gallbladder	Gallbladder unsuspicious; liver greenish	Granulocitic and histiocytic infiltrates; endothelial damage; severe cholestasis; centroacinary necrosis
4	Thickened gall bladder	Hepatomegaly	Severe cholestasis; no necrosis
5	Gall bladder hydropic, sludge	Bloody ascites; no signs of liver dystrophy	Severe necrosis; pronounced cholestasis; macrophage activation
6	Unsuspicious		Endothelialitis; bile duct-associated inflammation; similar to rejection reaction
	MRI: Irregular bile ducts		

**Fig 7:**
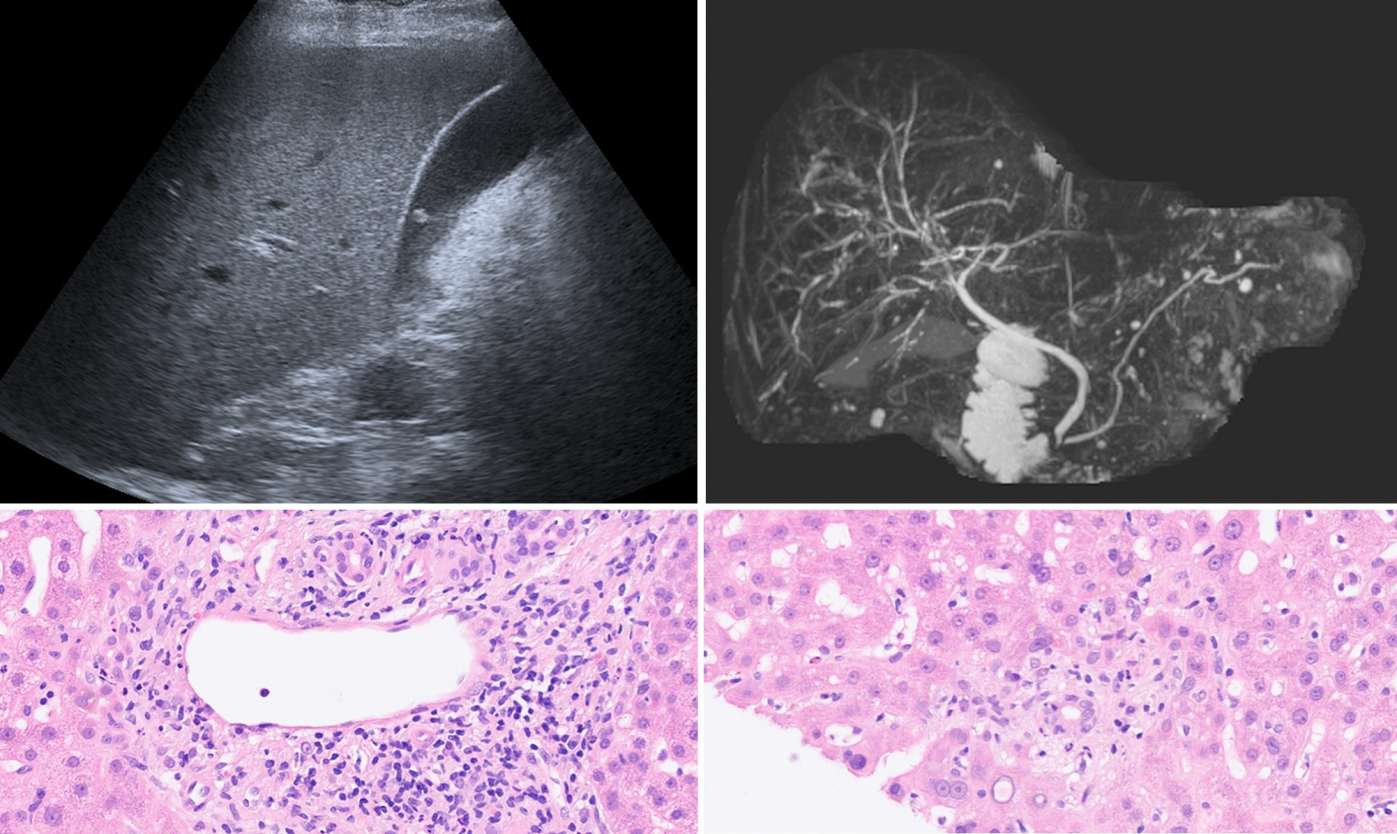
Patient of group 3 with persistent severe elevation of liver enzymes and GGT. (A) Whereas sonography was normal, (B) MRCP revealed irregular bile ducts leading to the suspected diagnosis of secondary sclerosing cholangitis (SSC). (C, D): Histologic specimen of the same patient showing signs of vascular- (C) and bile duct-associated (D) inflammation, which is typical for misguided immunoreaction like in rejection reaction. The diagnosis of SSC could not be confirmed.

One patient of group 3 with negative sonography (Fig 7A) received MRI of the liver, where irregular bile ducts were detected and interpreted as secondary sclerosing cholangitis (SSC, Fig 7B). Liver specimen revealed endothelialitis and inflammatory changes of bile ducts similar to rejection reaction, not typical for SSC though (Fig 7C and 7D).

## Discussion

COVID-19 is a highly contagious disease with extensive influence on public health. This study presents a representative patient cohort of a university hospital during the first outbreak of Corona Virus in Germany (Cohort 1) and additionally a non-representative patient cohort at the ICU (Cohort 2). In cohort 1, 37% of confirmed COVID-19 cases developed elevated liver enzymes, 83% of them only mildly. In previous studies, liver enzymes were elevated in 22% up to 53% of patients [1, 6, 7]. Huang et al. described elevation of AST in 62% of patients in the ICU compared to 25% of patients who did not require treatment in the ICU [8]. In our study, we show comparable results: Only one patient (1 / 80 = 1.3%) without elevated liver enzymes required treatment in the ICU, but 71.8% of those with mildly and all of those with severe elevation of liver enzymes. Whereas COVID-19 affects both men and women equally–exact 50% of the positively tested patients in cohort 1 were men and women–elevated liver enzymes seem to be more frequent in men, especially severe elevation. Furthermore, we see a significant increase in mortality between the different groups (group 1: 13.5%, group 2: 28.2%, group 3: 71.4%). It may be questioned if this is only due to the potential liver failure, since most of the deceased patients developed multi organ failure with the liver being only one part of it.

With respect to the ultrasound findings, most of the patients with only mildly elevated liver enzymes showed normal appearance of the liver (88.9%, 24 / 27) whereas 71.4% (5 / 7) of those with severely elevated liver enzymes had suspicious findings in ultrasound. We could identify two patterns of ultrasound findings:

1. Most frequently and pronounced in group 2, we found a variable combination of distinct thickening of the gallbladder, decreased echogenicity of the liver—in part with starry sky appearance or swelling of the liver—and ascites. These findings are typical, but not specific for acute hepatitis [9–11].
2. As a second, less frequent pattern, we found vascular complications in the liver: In four cases the Doppler signal of the hepatic artery was strongly reduced or not detectible, suggesting hepatic artery thrombosis. Two patients showed geographic zones of hypoechogenicity of liver parenchyma, a finding associated with hepatic infarction [12]. The second pattern was found especially in patients with severely elevated liver enzymes. All of these patients died the same day of the ultrasound examination.

These findings could be partially confirmed by autopsy. One case with sonographic signs of hepatitis showed granulocytic and histiocytic infiltrates in the liver. Necrosis was seen in both patients with signs of hepatitis and in one patient with geographic zones of hypoechogenicity. In one case, obliteration of hepatic artery ramifications was detected. Unfortunately, the hepatic artery was not processed histologically, thus hepatic artery thrombosis could not be proven. As endothelialitis and thus arterial and venous thromboembolism are frequently observed in severe COVID-19 cases [13, 14], this may be the underlying reason of vascular complications in the liver.

Four out of five autopsied patients had massive cholestasis with the only sonographic correlate being sludge filled and wall thickened gall bladder but no dilation of bile ducts. Laboratory findings of cholestasis and biliary sludge are common findings in critically ill patients [15, 16] and thus not specific for COVID-19. In addition, the sonographic findings of gallbladder wall thickening and cholestasis are very unspecific and may also occur in conditions not associated with primary liver damage.

Most striking is, however, another histologic finding: Three out of five autopsies showed macrophage activation, the histopathologic correlate of secondary hemophagocytic lymphohistiocytosis (sHLH). Leading to multi organ failure due to excessive cytokine secretion, sHLH is a hyperinflammatory syndrome most frequently triggered by viral infections [17]. sHLH was described as a rare but severe complication also in COVID-19 patients in a late phase of the disease when Coronavirus-RNA is mostly not detectible anymore and systemic hyperinflammation mimics ongoing sepsis [18]. Frequently elevated d-dimer and the above mentioned thromboembolisms can also be explained in this context, as they are manifestations of defective procoagulant-anticoagulant balance [19]. Even though our patients didn’t meet all the criteria for sHLH, macrophage activation seems to be a frequently occurring complication in our institution—most likely due to our institution being a university hospital with many transferred patients from other hospitals.

As a summary of the above findings the 68-year-old patient described in Fig 7 may be interesting. Although he survived COVID-19, liver enzymes and especially GGT increased up to his discharge against medical advice. Unsuspicious in sonography, magnetic resonance cholangiopancreatography (MRCP) revealed irregular bile ducts and suspected diagnosis of SSC was made. Liver specimen finally depicted inflammatory changes of the endothelium, bile ducts and sinusoids not fitting to the suspected diagnosis of SSC but similar to changes seen in misguided immune response e.g. in cases of rejection reaction following liver transplant.

This study has limitations: First, as a retrospective single-center study its generalizability is limited and it underlies a selection bias. Furthermore, since 14 patients of Cohort 2 were also included in Cohort 1, statistical power of Cohort 2 may be affected when removing them from the analysis. Second, only for a few patients, histologic correlation was available. Third, we only took the highest measured value of ALT for group assignment. Thereby different courses of liver enzyme elevation remain unconsidered.

In conclusion, in most cases of COVID-19 elevated liver enzymes cause little concern, as they are only mild to moderate. In these cases ultrasound is usually unremarkable. In severe cases though, especially at ICU, sonography can reveal vascular, cholestatic or inflammatory complications in the liver, which are associated with high mortality. It remains however unclear, whether these changes are caused by SARS-CoV-2 itself or by secondary hyperinflammation. Further studies are required to clarify the role of novel Coronavirus in acute liver damage.

## Supporting information

S1 Data Cohort 1. (XLSX)

S2 Data Cohort 2. (XLSX)
